# How have COVID‐19 stringency measures changed scholarly activity?

**DOI:** 10.1111/nyas.14767

**Published:** 2022-03-21

**Authors:** Kim M. Caudwell, Alessandro Soranzo, Lee Wei Lim, Luca Aquili

**Affiliations:** ^1^ College of Health & Human Sciences Charles Darwin University Darwin Northern Territory Australia; ^2^ Department of Psychology, Sociology & Politics Sheffield Hallam University Sheffield UK; ^3^ Neuromodulation Laboratory, School of Biomedical Sciences, Li Ka Shing Faculty of Medicine University of Hong Kong Hong Kong China

**Keywords:** COVID‐19, research productivity, scientists, global burden of disease, government restrictions

## Abstract

Government restrictions to the movement of people due to the COVID‐19 pandemic have had a wide range of effects on scientific activity. Here, we show that during the pandemic there has been a reduction in the number of registered non‐COVID‐19 clinical trials. Furthermore, using the Oxford COVID‐19 Government Response Tracker Stringency Index (SI) as an indicator of COVID‐19–related workplace adjustment (e.g., restrictions on gatherings, workplace closures, and stay‐at‐home orders), we demonstrate that this drop in clinical trial registration has been greater in countries with a higher SI. This could have significant consequences for the discovery of treatments that are required to reduce the global burden of disease.

## Graphical abstract

Government restrictions to the movement of people due to the COVID‐19 pandemic have had a wide range of effects on scientific activity. Here we show that during the pandemic, there has been a reduction in the number of registered non‐COVID‐19 clinical trials, which could have significant consequences for the discovery of treatments that are required to reduce the global burden of disease.

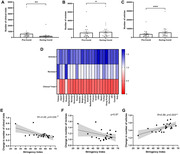

Workforce adjustments due to the COVID‐19 pandemic have had profound effects on academics, who are already beleaguered by the changing employment landscape. During the pandemic, an estimated 650,000 academics in the United States and 17,000 in Australia have lost their jobs, and many more have been affected by casualization.^1^, ^2^, ^3^ Additionally, the availability of start‐up funds for early career scientists has diminished, threatening the supply chain for continued research capability.^4^ The entire research community globally has been affected by COVID‐19, from graduate students and postdoctoral researchers^5^ to female scientists, especially those with children and those working in wet labs.^6^, ^7^, ^8^ At the same time, publications related to COVID‐19 have understandably increased. Moreover, the shorter median time from submission to acceptance for COVID‐19 papers compared to other diseases (such as Ebola and cardiovascular disease) has potentially impacted the quality of the review process.^9^ Similarly, as the international research focus has shifted to the pandemic, an estimated 80% of non‐COVID‐19‐related clinical trials have been stopped due to a combination of social distancing, lockdowns, and the prioritization of COVID‐19 research.^10^

Although COVID‐19 presents significant challenges in relation to the containment of the global pandemic, adjustments to the research environment stemming from COVID‐19 responses may have far‐reaching effects on reducing the global burden of disease (GBD) through the timely evaluation of new treatments. Adding urgency to the GBD are the challenges faced by individuals due to the impact of lockdown. For example, physical inactivity—linked to nearly 10% of the GBD—has increased to a greater extent in countries implementing more stringent measures in response to COVID‐19.^11^, ^12^

To better quantify the effects of the COVID‐19 stringency measures, such as social distancing restrictions, on research productivity, we conducted a web search using PubMed and ClinicalTrials.gov to compare the scholarly activity before and since the emergence of COVID‐19; these two web platforms were chosen because of the extensive coverage of biomedical topics and of being primary sites for registering clinical trials, respectively.

To ascertain the impact of stringency measures, we used the Oxford COVID‐19 Government Response Tracker^13^ Stringency Index (SI) as an indicator of COVID‐19–related workplace adjustment (e.g., restrictions on gatherings, workplace closures, and stay‐at‐home orders).

We expected that scholarly activities would change as a result of social restriction measures implemented by different countries, specifically those activities requiring researcher‐to‐participant involvement. We examined three forms of scholarly activity: (1) clinical trials (including randomized controlled trials (RCTs)), which involve substantial face‐to‐face contact with patients and participants; (2) reviews (including systematic reviews), which do not involve face‐to‐face contact; and (3) research articles (including opinion pieces, letters, commentaries, and studies in nonclinical contexts), which do not necessarily involve substantial face‐to‐face contact. We hypothesized that social distancing and lockdown restrictions have reduced the number of clinical trials, and increased the number of reviews/research articles, and that these effects correlate with a country's SI.

## Scholarly activity before and during COVID‐19

The data collection was conducted in PubMed and ClinicalTrials.gov and used a single‐query search encompassing the deadliest diseases (e.g., cardiovascular, stroke, dementia, cancer, smoking, and suicide) and chronic conditions (e.g., diabetes, addiction, depression, and anxiety) but excluding COVID‐19–related papers (details of our approach are included as supplementary methods in File S1, online only). To measure changes in research productivity before and during COVID‐19, we compared the periods January–July 2019 with January–July 2021. Given the varying time between trial registrations and publication of results, we considered changes in the number of clinical trial registrations (not publication) using ClinicalTrials.gov. We used the Oxford COVID‐19 Government Response Tracker to identify the countries’ SI, which were included with our PubMed and ClinicalTrials.gov search. Finally, we ran correlation analyses^a^ between the changes in scholarly activity and a country's SI.

## Reduction in clinical trials during COVID‐19

**Figure 1:**
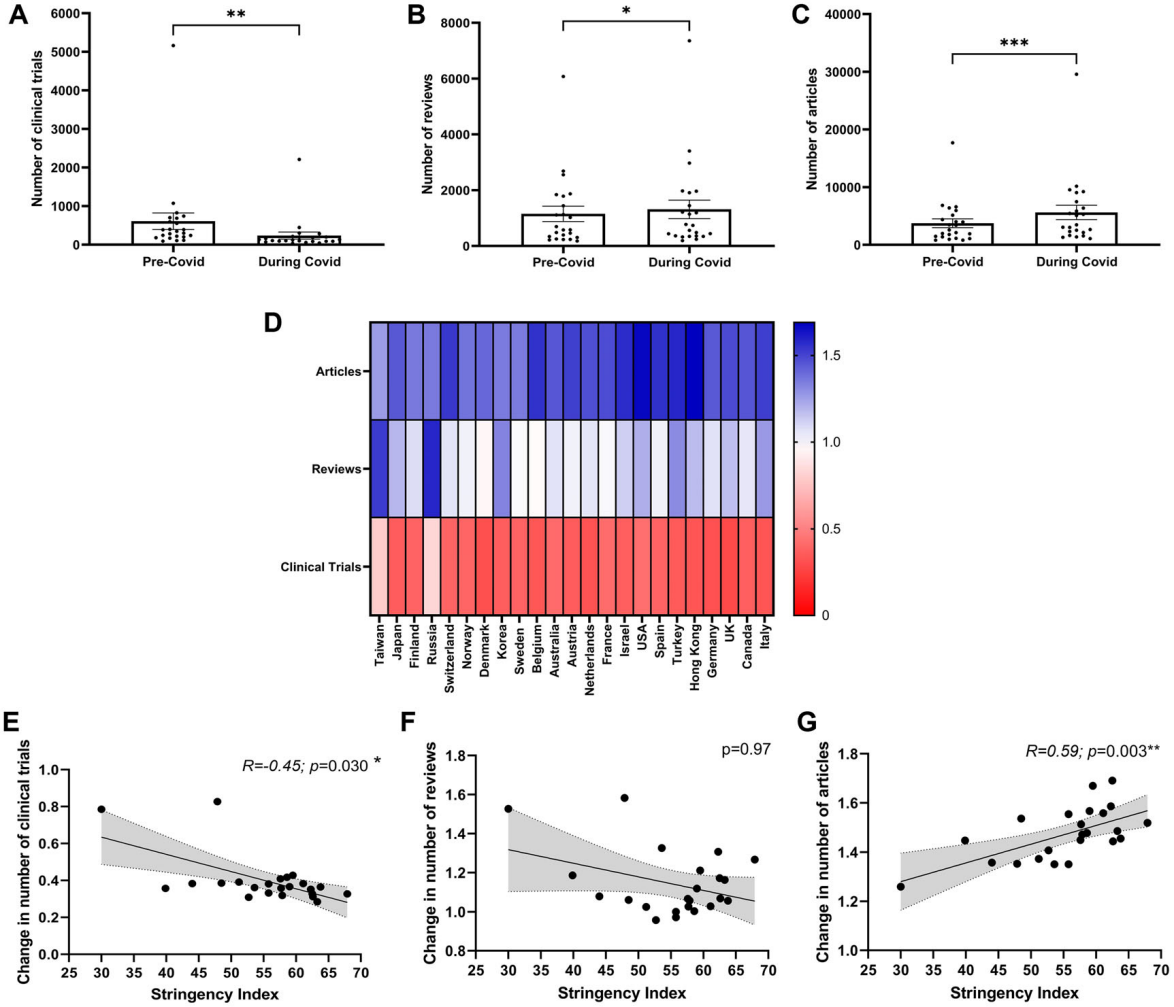
Comparisons between research productivity before and during COVID‐19 for (A) clinical trials, (B) reviews, and (C) articles/opinion pieces/letters/commentary. Statistical tests were conducted using nonparametric alternatives to paired sample *t*‐test for data not normally distributed or with small sample sizes (Wilcoxon matched‐pair signed‐rank). (D) Heatmap of the relationship between countries with differing SI (left side: lowest stringency [e.g., Taiwan], right side: highest stringency [e.g., Italy]) and changes in research productivity, as measured by registered clinical trials (including RCTs), published reviews, and articles/opinion pieces/letters/commentary. Change in productivity was measured by dividing the total number of research outputs (keywords in PubMed/ClinicalTrials.gov included: cardiovascular, stroke, dementia, cancer, smoking, suicide, diabetes, addiction, depression, and anxiety) for a given country in the period between January and July 2021 by the same period in 2019. A score of 1 indicates no change (white color), a score toward 0 indicates a decrease in research productivity (red color), and a score toward 1.5 indicates an increase in productivity (blue color). Nonparametric Spearman's rho correlation between the country's SI and changes in research productivity by (E) clinical trials, (F) reviews, and (G) articles/opinion pieces/letters/commentary. ^*^ *P* ≤ 0.05; ^**^ *P* ≤ 0.01; ^***^ *P* ≤ 0.001.

We observed a significant reduction (*P* < 0.001, Fig. 1A) in the number of registered clinical trials during the COVID‐19 period, compared to a corresponding pre–COVID‐19 period. These findings are consistent with a statement by the U.S. National Institutes of Health^10^ indicating that approximately 80% of non‐COVID‐19 trials were stopped during the pandemic. In contrast, there has been a significant increase in the number of reviews (*P* ≤ 0.001, Fig. 1B) and research articles (*P* ≤ 0.001, Fig. 1C) during the same time. Additional analyses (see File S1, online only) suggest that this increase has been steady since at least 2016; hence, pre–COVID‐19. On the other hand, the number of registered clinical trials before COVID‐19 was stable or slightly increased, while there was a greater reduction in clinical trials during COVID‐19 between 2020 and 2021.

## Decline in clinical trial registrations compensated increase in other scholarly outputs

Figure 1D illustrates how countries with different SIs have been affected in terms of scholarly activity during COVID‐19. Countries with low stringency measures (e.g., Taiwan, Japan, and Finland) continued to register clinical trials and publish reviews and research articles at a relatively unchanged rate during the pandemic, whereas high stringency countries (e.g., the UK, Canada, and Italy) showed a substantial drop in clinical trial registrations that was somewhat compensated by an increase in research article publications.

Spearman's rho correlation analyses of 23 countries found a negative correlation (*r_s_ = *−0.45, *P* = 0.030; Fig. 1E) between a country's SI and the change in the number of registered clinical trials, as measured by dividing output in 2021 by 2019. We also found the changes in the number of related articles (e.g., opinion pieces, letters, commentary, and research articles) were positively associated with a country's SI (*r_s_ = *0.59, *P* = 0.003; Fig. 1G). No significant association was found between a country's SI and the number of review articles (*P* = 0.97, Fig. 1F). To assess whether the lack of significance supports an absence of an effect, we conducted Bayesian rank‐based hypothesis testing;^14^ the Bayes factor indicated the likelihood of the hypothesis that the number of review articles and a country's SI are uncorrelated is 3.83 times higher than the hypothesis that the two are correlated.

We also conducted a control analysis to test whether the positive correlation between a country's SI and a change in the number of articles was unrelated to the social restrictions. For this purpose, we analyzed the association between a country's SI and the published outputs between 2017 (Jan–July) and 2019 (Jan–July), when there were no social restrictions: no significant association was found between a country's SI and the number of published articles during this time (*P = *0.70, see results in File S1, online only). Bayesian rank‐based hypothesis testing^14^ indicated the likelihood that the hypothesis that the two variables are not correlated is 3.9 higher than the hypothesis that they are correlated.

## What all this means

Clinical trials, particularly RCTs, are widely considered to provide the most trustworthy evidence for the effectiveness of treatments and interventions.^15^, ^16^, ^17^ Work closures and stay‐at‐home orders during COVID‐19 have delayed ongoing clinical trials^10^ and the commencement of new trials, as demonstrated by our analyses. At the same time, we observed that a country's SI was positively correlated with the number of published research articles. We speculate that researchers living in countries with high levels of social restrictions and reduced RCT productivity may have compensated by publishing more opinion pieces, letters, commentaries, and research articles. This would have been possible because social restrictions likely had a less impact on these types of research activities. The lack of an association between published reviews and a country's SI appears to indicate that the shift in publications has not extended to review‐type articles, perhaps because review articles may take longer to write up and publish.

Our work has several caveats that need to be considered. First, our selection of the time periods for research productivity during COVID‐19 in published reviews and articles may mean that the data in a proportion of these publications would have likely been collected before the COVID‐19 pandemic. However, this does not influence the findings related to clinical trials as we only measured trial registration in this period. Second, our analysis was limited to 23 countries; we did not include countries that did not generate a statistically meaningful number of publications or registrations. The countries selected in our analysis included major academic institutions with significant output.

## The road ahead

The prioritization of COVID‐19–related research, medical efforts, and stringency measures have inevitably had several unintended, yet detrimental, consequences. These include delayed treatment and diagnosis of cancers,^18^ decreased number of suspected stroke presentations at hospitals,^19^ and worsening psychiatric symptoms in individuals with dementia due to social isolation.^20^ ^,21^ With the increasing number of people being vaccinated against COVID‐19 and as societies reopen, we hope the number of clinical trials will gradually return to pre–COVID‐19 levels. This is far from certain, however, given the impact of the loss of valuable resources, including workforce and revenue incurred by many universities and research centers. It also remains unclear how government responses will impact research productivity in subsequent (potential) waves of COVID‐19. Our findings suggest that restrictions need to be carefully considered in relation to their effects on research productivity, especially where such research aims to reduce the disease burden of conventional illnesses, that will continue to affect populations long after COVID‐19 has been effectively controlled.

## Author contributions

L.A. conceived the study. K.M.C. and L.A. developed the methodology. K.M.C., A.S., and L.A. analyzed the data. K.M.C., A.S., L.W.L., and L.A. wrote the manuscript.

## Competing interests

The authors declare no competing interests.

### Peer review

The peer review history for this article is available at: https://publons.com/publon/10.1111/nyas.14767.

## Supporting information

Supplementary material

## Footnotes

a Spearman's rho correlation was used to determine the influence of outliers.
