# Supplementary material for: How have COVID‐19 stringency measures changed scholarly activity?
Source: Ann N Y Acad Sci. 2022 Mar 21;1513(1):5–9. doi: 10.1111/nyas.14767 (PMC9115337; doi:10.1111/nyas.14767)

**Supplementary Methods**

1. Supplementary methods (Search string and Selection of countries with different stringency indexes)
2. Supplementary Results (Control analyses (1) and (2))

**Search string in PubMed and ClinicalTrials.gov**

The following search string was used to retrieve 1) research articles, opinion pieces, letters, commentary, and other forms of publications not including reviews, systematic reviews, clinical trials and RCTs in PubMed; 2) reviews and systematic reviews in PubMed; and 3) clinical trials in ClinicalTrials.gov (including RCTs).

(("depression"[Title/Abstract] OR "anxiety"[Title/Abstract] OR "schizophrenia"[Title/Abstract] OR "addiction"[Title/Abstract]) OR "memory"[Title/Abstract] OR "cognitive"[Title/Abstract] OR "cardiovascular"[Title/Abstract] OR "dementia"[Title/Abstract] OR "Alzheimer's"[Title/Abstract] OR "cancer"[Title/Abstract]) OR "stroke"[Title/Abstract] OR "diabetes"[Title/Abstract] OR "reaction time"[Title/Abstract]) OR "smokers"[Title/Abstract] OR "cerebrovascular"[Title/Abstract] OR "suicide"[Title/Abstract] ) NOT (mice)) NOT (rats)) NOT (survey)) NOT (questionnaire)) NOT (covid)) AND (name of one of the 23 countries[Affiliation])) AND (("either 2019/01/01 OR 2021/01/01"[Date - Publication] : "2019/07/31 OR 2021/07/31"[Date - Publication]))

The above search was performed for each of the correlational analyses both before 01/01/2019 to 31/07/2019 and during COVID-19 (01/01/2021 to 31/07/2021). We excluded terms such as “mice”, “rats” and “survey” as these would likely involve less face-to-face contact. We also excluded any research publications that contained the term “COVID”, as we already know there was a substantial increase in publications in relation to this topic. We chose the 01/01/2021 to 31/07/2021 period as most countries had already undergone the first “wave” of COVID-19 and many were undergoing a second wave, during which we assumed COVID-19 policies (i.e., as measured by the country’s stringency index) would impact research productivity.

(1), (2), and (3) were selected in PubMed using the tick box options as shown below.


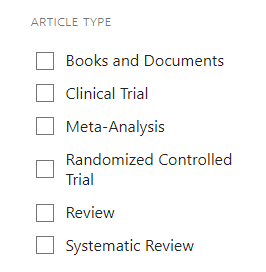


For clinical trials, we used <https://clinicaltrials.gov/> to measure research productivity during these periods according to trial registration. We took the date on which the study record was first available on ClinicalTrials.gov, regardless of status (e.g., recruiting, completed, ongoing, unknown).

**Selection of countries by stringency index**

Data was obtained from the Oxford COVID-19 Government Response Tracker (OxCGRT) which is freely available at <https://ourworldindata.org/grapher/covid-stringency-index>.

The COVID-19 stringency index (SI) is made up of nine different measures including school closures, workplace closures, cancellation of public events, restrictions on public gatherings, closures of public transport, stay-at-home requirements, public information campaigns, restrictions on internal movements, and international travel controls. These indicators were measured on an ordinal scale and then summed and rescaled to a score ranging from 0 (very low stringency) to 100 (very high stringency).

A score is provided almost daily for each country. We calculated the average scores from the first day of the introduction of COVID-19 measures (21/1/2020 for most countries) until 31/7/2021.

The selection of countries in our analyses was based on two criteria. The first and most important criterion was that the country had enough research productivity within the 7-month period before and during COVID-19. We chose an arbitrary minimum of at least 100 research outputs (for correlational analyses 1, 2 and 3 [see page 2 of this document]). A country with fewer than 100 outputs was excluded from our analyses. We measured the change in productivity as a proportion (e.g., outputs in year X/outputs in year X-1).

The second criterion was based on the selection of countries within 95% of all recorded SIs to exclude outliers (China was excluded according to this criterion). The 23 countries that met both criteria1 and 2 had an SI between 30 (Taiwan) to 67.9 (Italy).

**Control analyses (1): overall increase in the number of reviews and articles, but a decline in the number of clinical trials during COVID-19**

To control that the change in the number of academic outputs is related to the COVID-19 and not to a general trend, we compared the number of outputs published from 2016 to 2021 in the same 7-month period [January 1^st^ to July 31^st^ and using the same keywords). As shown in the figure, a clear trend related to COVID-19 emerges for clinical trials but not for reviews. Whilst the number of clinical trials in 2021 was the lowest recorded since (at least) 2016, the number of reviews show a steady increase from 2016 until 2021.


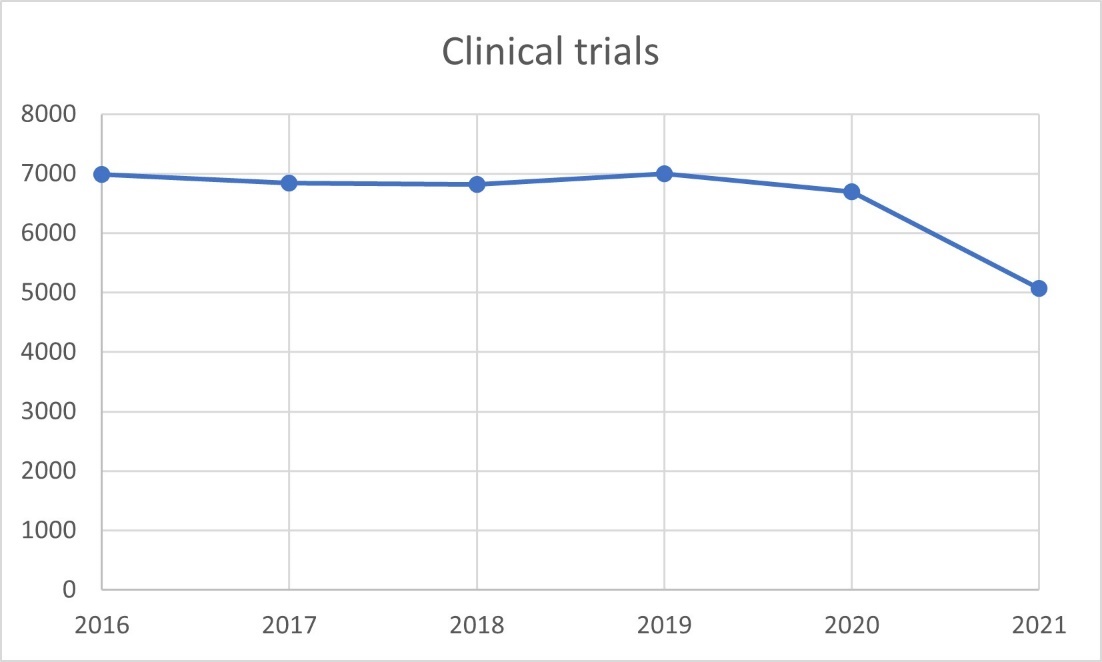


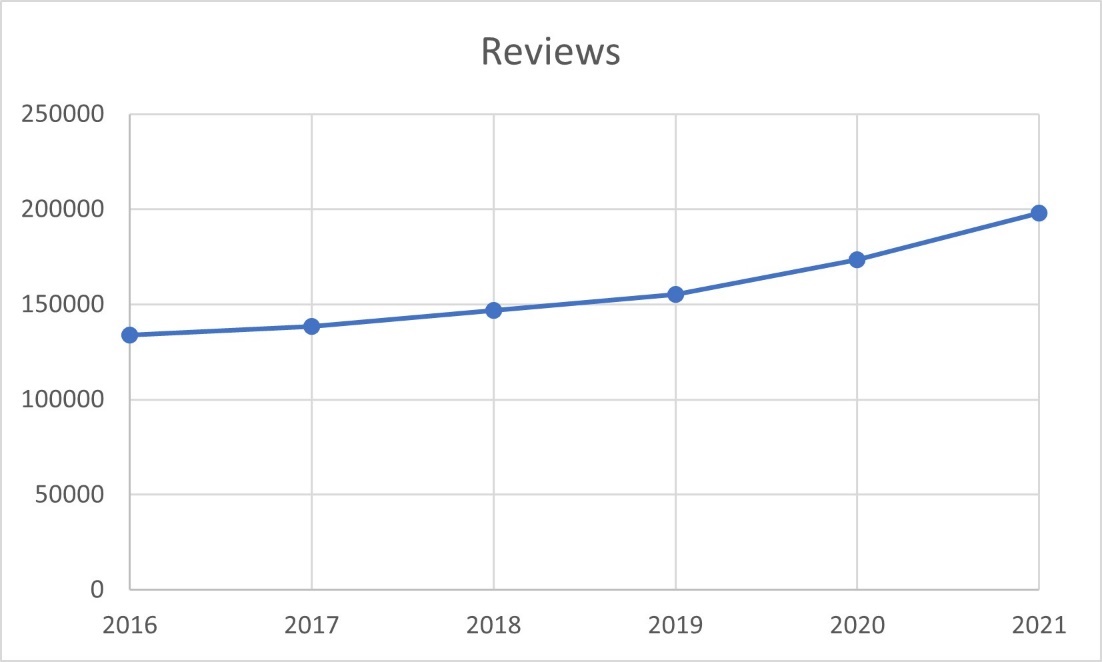


*In our current analysis of clinical trials, we looked at changes in registration not publication.

**Control analyses (2): significant positive correlation between research article productivity and country’s SI** **is specific to the COVID-19 period**

To control that the positive correlation between the stringency index and the change in the number of opinion pieces, letters, commentary, and research articles (Fig. 1G) is indeed associated to the COVID-19 pandemic, we repeated the same search considering the Jan-July periods in 2017 and 2019 using the same search terms.

A Spearman’s rho correlation does not show an association (p = 0.70) between country’s SI and research productivity (see scatter plot below). These results confirm that the COVID-19 social restrictions altered the publication of opinion pieces, letters, commentary, and research articles likely due to the social restriction policies, as measured by the country’s SI.


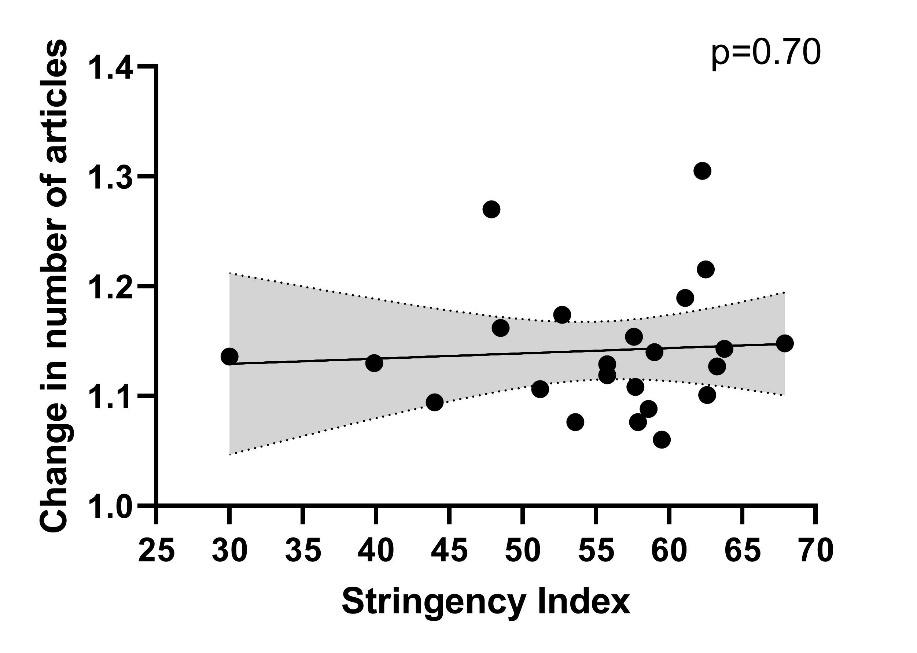

Supplement: Supplementary file 1 — Supplementary material [file NYAS-1513-5-s001.docx]
